# Repeated Perioperative Airway Management in Odontogenic Deep Neck Space Infection Managed Clinically as Ludwig’s Angina: An Anaesthesia- and Intensive Care Unit-Focused Case Report

**DOI:** 10.7759/cureus.112937

**Published:** 2026-07-18

**Authors:** Saif M AL-Shehab, Refaat A AL-Shehab

**Affiliations:** 1 Anesthesia and Critical Care, Prince Hamzah Hospital, Amman, JOR; 2 Anesthesia, AL-Abdali Hospital, Amman, JOR

**Keywords:** anesthesia, c-mac video laryngoscopy, deep neck space abscess, difficult airway intubation, extubation in icu, ludwig angina, odontogenic infection, streptococcus agalactia, tracheal deviation to the left

## Abstract

Ludwig's angina and odontogenic deep neck space infections represent high-risk scenarios for anaesthesiologists, as airway compromise may develop despite initially preserved spontaneous breathing. We report a 41-year-old woman with no relevant comorbidities who presented with a five-day history of odontogenic right-sided neck swelling, fever, odynophagia, dysphagia, muffled voice, and mild discomfort in the supine position. She was breathing spontaneously on room air with an oxygen saturation of 98%. CT neck demonstrated a right submandibular collection measuring 3 × 0.6 cm, multiple right sternocleidomastoid (SCM) intramuscular collections, and bilateral deep cervical lymphadenopathy. Leftward tracheal deviation was identified on clinical imaging review. On Day 0, emergency drainage was performed under general anaesthesia. C-MAC video laryngoscopy revealed a Cormack-Lehane grade 1 view; the trachea was intubated on the first attempt with rocuronium 1.2 mg/kg, without complication. The patient was maintained intubated in the ICU for approximately 24 hours and extubated following cuff-leak testing, corticosteroid pre-treatment, and multidisciplinary team reassessment. On Day 5, a second general anaesthetic was required for repeat drainage; again, grade 1 direct laryngoscopy, first-attempt intubation, and immediate post-procedure extubation were achieved. The pus culture grew *Streptococcus agalactiae*, susceptible to levofloxacin, vancomycin, and amoxicillin/clavulanate. Total antimicrobial therapy comprised seven days of intravenous therapy followed by 14 days of oral amoxicillin/clavulanate, for a total course of 21 days. The patient was discharged on Day 13 in stable condition. This case highlights that the absence of stridor or hypoxaemia does not eliminate significant airway risk in deep neck infection. Muffled voice, pain-limited oral assessment, supine discomfort, multi-compartment collections, SCM involvement, and tracheal deviation require structured difficult-airway planning, surgical airway readiness, planned extubation, and repeat airway reassessment for subsequent procedures.

## Introduction

Ludwig's angina is classically described as a rapidly progressive bilateral infection involving the floor of the mouth and the submandibular, sublingual, and submental spaces, most commonly arising from odontogenic sources. Airway compromise - driven by progressive tongue elevation, supraglottic oedema, and anatomical distortion - is the principal cause of morbidity and mortality [1]. Management priorities include airway protection, broad-spectrum intravenous antimicrobial therapy, source control, and surgical drainage when abscess formation is identified [1,2].

For anaesthesiologists, Ludwig's angina and related deep neck space infections represent some of the most hazardous airway scenarios encountered in perioperative practice. A patient who appears clinically stable while awake and upright may deteriorate rapidly after sedation, induction of anaesthesia, supine repositioning, or surgical manipulation. Anaesthetic airway management must, therefore, be individualised and must incorporate a primary airway plan, backup strategies, rescue oxygenation pathways, and immediate readiness for invasive front-of-neck access [1,3,4].

We report a 41-year-old woman with an odontogenic deep neck space infection managed clinically as Ludwig's angina, complicated by a right submandibular abscess, multiple right sternocleidomastoid (SCM) intramuscular collections, clinician-confirmed leftward tracheal deviation, and clinically documented sepsis without shock. The case involved two separate general anaesthetics, repeated operative drainage, and structured perioperative airway decision-making throughout. This report adheres to the CARE case report guidelines [3].

## Case presentation

A 41-year-old woman presented to a tertiary hospital in Jordan on 02 June 2026 with a five-day history of progressive right-sided neck swelling. Her height was 165 cm, weight 72 kg, and body mass index 26.4 kg/m². She was an active smoker with a 15-pack-year history and denied alcohol consumption. She had no known diabetes mellitus, immunosuppression, steroid therapy, chemotherapy, HIV infection, pregnancy, postpartum state, regular medications, or drug allergy. She had no prior anaesthesia exposure and no known difficult-airway history. Baseline functional status was fully independent. She was classified as American Society of Anesthesiologists (ASA) physical status II-E [4].

Dental pain had been present for approximately five days prior to admission; the right second molar was suspected as the odontogenic source. Presenting symptoms included fever (38.2°C), right-sided neck swelling, odynophagia, dysphagia, muffled voice, mild discomfort when lying flat, and reduced oral intake. She was able to swallow saliva with pain. She denied dyspnoea and stridor. No fixed trismus was documented; however, formal oral examination was limited by pain-restricted mouth opening. The Mallampati class could not be assessed. Reliable assessment of floor-of-mouth swelling, tongue elevation, and sublingual involvement was not possible.

Clinical examination demonstrated right-sided submandibular swelling with erythema, warmth, tenderness, and fluctuation. Neck range of motion, thyromental distance, and sternomental distance were normal. There was no secretion burden and no sign of airway obstruction at rest. The anaesthesia note documented difficulty speaking, with a Glasgow Coma Scale (GCS) score of 15/15 recorded. Vital signs in the anaesthesia note were blood pressure 132/86 mmHg, heart rate 109 beats/min, respiratory rate 22 breaths/min, and SpO₂ 98% on room air. Vital signs varied across documentation; the patient remained spontaneously breathing on room air throughout without stridor or severe respiratory distress. Laboratory investigations are summarised in Table 1.

**Table 1:** Initial Laboratory Investigations WBC = white blood cell count; RBC = red blood cell count; MCV = mean corpuscular volume; MCH = mean corpuscular haemoglobin; MCHC = mean corpuscular haemoglobin concentration; RDW = red cell distribution width; MPV = mean platelet volume; H = above reference range; L = below reference range; HbA1c = glycated haemoglobin; CRP = C-reactive protein.

Parameter	Result	Reference Range	Flag
Complete Blood Count			
WBC (×10⁹/L)	18.27	4.0–11.0	H
RBC (×10¹²/L)	4.08	4.1–5.3	L
Haemoglobin (g/dL)	8.5	12–16	L
Haematocrit (%)	27.8	35–47	L
MCV (fL)	68.1	80–96	L
MCH (pg)	20.8	28–33	L
MCHC (g/dL)	30.6	33–36	L
RDW (%)	18.8	11.0–16.0	H
Platelet count (×10⁹/L)	191	150–450	Normal
MPV (fL)	10.5	6.0–11.0	Normal
Neutrophils (%)	86.1	40–80	H
Lymphocytes (%)	10.1	20–40	L
Monocytes (%)	3.5	2–10	Normal
Eosinophils (%)	0.2	1–6	L
Biochemistry and Coagulation			
Renal function	Normal	Within normal limits	-
Electrolytes	Normal	Within normal limits	-
Liver function tests	Normal	Within normal limits	-
Random glucose	Normal	Within normal limits	-
Coagulation profile	Normal	Within normal limits	-
Unavailable Tests			
Lactate	Not measured	Lactate kit unavailable at institution at that time	-
C-reactive protein (mg/L)	Not available	-	-
Procalcitonin (ng/mL)	Not available	-	-
HbA1c (%)	Not available	-	-

Lactate was not measurable because the lactate kit was unavailable at the institution at that time. C-reactive protein, procalcitonin, and HbA1c were not available. Sepsis was clinically identified by the treating team, with the deep neck abscess as the attributed source. There was no hypotension, no vasopressor requirement, and no documented organ dysfunction.

**Figure 1:**
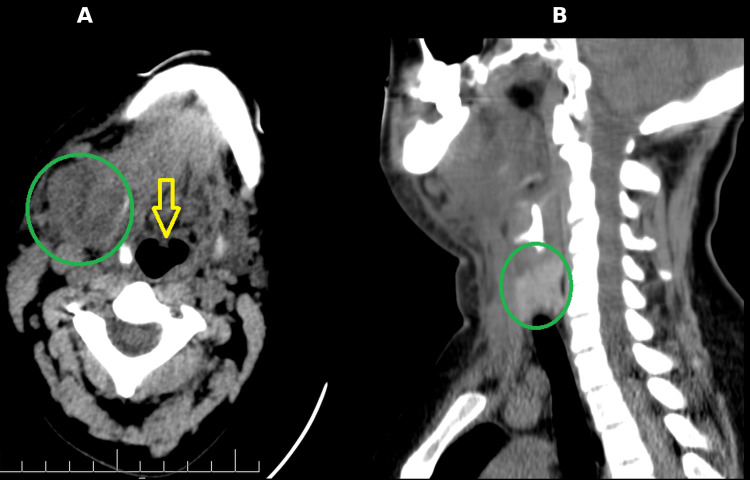
Preoperative CT Neck Figure 1A (axial): Green circle - right submandibular inflammatory mass and collection medial to the right submandibular gland with sternocleidomastoid involvement. Yellow arrow - clinician-confirmed leftward tracheal deviation. Figure 1B (sagittal): Green circle - right-sided deep neck space collection within the sternocleidomastoid region. All images are fully anonymised; patient identifiers, accession numbers, timestamps, and provider names have been removed.

CT neck demonstrated an enlarged heterogeneous right submandibular gland without intraglandular collection; a collection measuring 3 × 0.6 cm medial to the right submandibular gland; multiple collections within the right sternocleidomastoid muscle; and bilateral deep cervical lymphadenopathy more prominent on the right. The nasopharyngeal space was patent and the thyroid gland was unremarkable. The CT report did not include objective airway diameter measurements. Leftward tracheal deviation was identified on clinical imaging review and confirmed with the radiology team (Figures 1A, 1B).

No mediastinal extension, gas formation, or necrotising fasciitis signs were identified on CT imaging. The working diagnosis was odontogenic deep neck space infection managed clinically as Ludwig's angina, complicated by a right submandibular abscess, multiple right SCM intramuscular collections, confirmed leftward tracheal deviation, and clinically documented sepsis without shock.

First anaesthetic and airway management - Day 0

The patient was transferred to the operating room for emergency incision and drainage. Despite the absence of stridor, dyspnoea, or drooling, the airway was assessed as potentially difficult based on the following features: muffled voice, documented difficulty speaking, pain-limited oral examination, dysphagia, odynophagia, mild supine discomfort, progressive submandibular infection with CT-documented SCM involvement, and confirmed leftward tracheal deviation.

The primary airway plan was tracheal intubation. The backup plan was tracheostomy. Emergency front-of-neck access by cricothyrotomy was considered a rescue pathway. The difficult-airway cart, flexible bronchoscope, and tracheostomy equipment were all available and prepared. The otolaryngology surgeon was present in the operating room throughout the airway establishment.

Awake tracheal intubation could not be performed, as the procedure was classified as an emergency; therefore, the team elected to perform controlled airway assessment and intubation in the operating room with immediate otolaryngology backup and surgical airway readiness. This was the strategy individualised to this specific clinical context and should not be interpreted as evidence of general superiority over awake techniques. Awake tracheal intubation is supported as the preferred technique for anticipated difficult airways by the Difficult Airway Society guidelines and should be considered whenever indicated, feasible, and available [2,5].

The patient was maintained in spontaneous ventilation and preoxygenated with supplemental oxygen at 8 L/min using deep tidal breathing. Induction was achieved with midazolam 1.5 mg, fentanyl 150 μg, and propofol 150 mg, administered sequentially. No topical airway anaesthesia was applied. Airway assessment was performed using a C-MAC video laryngoscope with a size three Macintosh blade, which immediately revealed a Cormack-Lehane grade 1 laryngoscopic view. Rocuronium 1.2 mg/kg was then administered, and the trachea was intubated on the first attempt with a cuffed 7.0-mm endotracheal tube without requirement for a stylet or bougie. Tracheal intubation was confirmed by continuous waveform capnography and bilateral chest auscultation. Time from induction to intubation was approximately 50 seconds. The lowest recorded SpO₂ was 99%. No airway bleeding, pus contamination, pulmonary aspiration, desaturation, bradycardia, hypotension, or airway complication occurred.

Excision and drainage of the right submandibular collection was performed via a right submandibular incision. Thick yellowish pus was drained; the volume was not measured. No necrotic tissue was identified, and debridement was not performed. A surgical drain was placed within the abscess cavity following evacuation of purulent material. Dental extraction was not performed at this operation; maxillofacial surgery outpatient follow-up was arranged for definitive dental management.

Anaesthesia duration was approximately one hour and 30 minutes; surgical duration was approximately one hour and 15 minutes. Anaesthesia was maintained with sevoflurane in volume-controlled ventilation with a peak airway pressure of 16 cmH₂O. The patient received 1,200 mL of Ringer's lactate intravenously. Blood loss was minimal. Urine output was 200 mL. No vasopressor was required. Intraoperative medications included dexamethasone 8 mg, hydrocortisone 100 mg, morphine 8 mg, paracetamol 1 gm, and nefopam hydrochloride 20 mg. Neuromuscular monitoring was not used; reversal was not administered as the patient remained intubated postoperatively. No intraoperative complication occurred.

ICU course and extubation

The patient was transferred intubated to the surgical ICU for airway protection, controlled mechanical ventilation, and close monitoring. Ventilation was delivered in synchronised intermittent mandatory ventilation (SIMV) mode: tidal volume 450 mL, respiratory rate 12 breaths/min, positive end-expiratory pressure (PEEP) 5 cmH₂O, pressure support 8 cmH₂O, FiO₂ 0.30, and inspiratory-to-expiratory ratio 1:2. ICU sedation and analgesia were maintained with propofol 200 mg/hour and remifentanil 8 μg/kg/hour. The patient was haemodynamically stable throughout and required no vasopressor support.

Intravenous antimicrobial therapy included levofloxacin 750 mg once daily and vancomycin (loading dose 1,750 mg, then 1,200 mg twice daily, adjusted according to trough levels). The total intravenous course was seven days (Day 0 to Day 6 inclusive).

Extubation was performed approximately 24 hours after initial surgery following reassessment by the chief anaesthesia consultant. A positive cuff-leak test was confirmed, a repeat bedside airway assessment was performed, and corticosteroids were administered before extubation. Both the anaesthesia and otolaryngology teams were present, and a reintubation plan was explicitly prepared. An airway exchange catheter was not used. The patient was extubated in the ICU to simple face-mask oxygen. There was no post-extubation stridor, respiratory compromise, laryngospasm, or requirement for reintubation during 24 hours of close monitoring. The Difficult Airway Society extubation guideline classifies such patients as having at-risk airways requiring a planned extubation strategy and post-extubation monitoring [6].

Second anaesthetic and repeat drainage - Day 5

On Day 5 (five days after the initial operation and four days after extubation), the patient returned to the operating room for drainage of small residual or newly developed collections. General anaesthesia was induced with midazolam 1.5 mg, fentanyl 100 μg, propofol 150 mg, and cisatracurium 14 mg. Direct laryngoscopy demonstrated a Cormack-Lehane grade 1 view. The trachea was intubated on the first attempt with a cuffed 7.0-mm endotracheal tube. No increased airway difficulty was encountered compared with the first anaesthetic. No desaturation, bleeding, or airway complication occurred. No new drain was inserted, and no repeat pus culture was sent. The patient was extubated immediately after the procedure without complication.

Microbiology and antimicrobial therapy

A pus specimen was collected on Day 0. The bacteriology final report was issued on Day 3 and identified *Streptococcus agalactiae* (Group B *Streptococcus*). Blood cultures showed no growth. Anaerobic culture showed no growth. Gram stain was not performed. Susceptibility testing demonstrated susceptibility to penicillin, amoxicillin/clavulanate, ceftriaxone, ciprofloxacin, levofloxacin, and vancomycin, and resistance to amikacin, clindamycin, erythromycin, and trimethoprim/sulfamethoxazole. No antimicrobial change was made after culture results, as the isolate was susceptible to agents already in use. At discharge, the patient received oral amoxicillin/clavulanate 500/125 mg three times daily for seven days, completing a total antimicrobial course of 21 days (seven days intravenous followed by 14 days oral). An infectious disease consultation was not documented.

Odontogenic deep neck infections are frequently polymicrobial [5]. Recovery of *S. agalactiae* is reported accurately for what was cultured; a single aerobic culture and the absence of Gram stain do not exclude a polymicrobial aetiology.

Follow-up and outcome

The patient remained in the ICU until Day 7 and was then transferred to the ward, where she remained until discharge on Day 13. Oral intake resumed approximately one day after extubation. Swallowing, pain, and neck swelling improved progressively. The drain was removed on the last day of the ICU stay. A serial complete blood count reportedly normalised, although the exact serial values were not available from the anaesthesia record.

No airway complication, pulmonary aspiration, haemodynamic instability, vasopressor requirement, or emergency surgical airway occurred during either anaesthetic exposure. Repeat operative drainage on Day 5 was required and is a recognised feature of complex deep neck infection management. The patient was discharged in stable condition with dental follow-up arranged by the maxillofacial surgery team. No recurrence was documented in the available records. Long-term follow-up data were not available. Table 2 presents the timeline of clinical events.

**Table 2:** Timeline of Clinical Events Day 0 = date of admission and first surgical procedure. OR = operating room; ICU = intensive care unit; ENT = otolaryngology; CBC = complete blood count; WBC = white blood cell count; Hgb = haemoglobin; SCM = sternocleidomastoid muscle; CT = computed tomography; C-MAC = video laryngoscope brand; C-L = Cormack-Lehane; ETT = endotracheal tube; SIMV = synchronised intermittent mandatory ventilation; I&D = incision and drainage; IV = intravenous; OD = once daily; TID = three times daily.

Time Point	Event
Day -5 to Day -1	Dental pain from right second molar; progressive right-sided neck swelling
Day 0 (Admission)	Right-sided neck swelling, fever 38.2°C; admitted under ENT as Ludwig's angina (ASA II-E); CBC: WBC 18.27×10⁹/L, neutrophilia 86.1%, Hgb 8.5 g/dL (microcytic hypochromic), platelets 191×10⁹/L; coagulation normal; lactate unavailable
Day 0	CT neck: 3×0.6 cm right submandibular collection, multiple right SCM collections, bilateral cervical lymphadenopathy; confirmed leftward tracheal deviation
Day 0 (OR)	ENT surgeon present; tracheostomy equipment, difficult-airway cart, and flexible bronchoscope prepared; C-MAC video laryngoscopy C-L grade 1; rocuronium 1.2 mg/kg; first-attempt intubation; 7.0-mm cuffed ETT; no complication
Day 0 (OR)	Right submandibular I&D; thick yellowish pus drained; surgical drain placed; pus sent for culture; sevoflurane maintenance; no vasopressors
Day 0 (ICU)	Transferred intubated to surgical ICU; SIMV ventilation; propofol 200 mg/h + remifentanil 8 mcg/kg/h; IV levofloxacin 750 mg OD + vancomycin commenced
Day 1 (ICU)	Extubated 24 h post-surgery; positive cuff-leak test; corticosteroids given; ENT and anaesthesia teams present; reintubation plan prepared; no post-extubation stridor or reintubation
Day 3	Bacteriology final report: *Streptococcus agalactiae*; susceptible to levofloxacin, vancomycin, amoxicillin/clavulanate; no antibiotic change
Day 5 (OR)	Second anaesthetic for repeat drainage; direct laryngoscopy C-L grade 1; first-attempt intubation; no complications; immediate extubation post-procedure
Day 6	IV antimicrobials completed - 7-day course
Day 7	Drain removed; transferred from ICU to ward; oral amoxicillin/clavulanate 500/125 mg TID was commenced
Day 13	Discharged on oral amoxicillin/clavulanate 500/125 mg TID × 7 days (total course: 21 days); dental follow-up arranged by maxillofacial surgery
Post-discharge	No recurrence in available records; long-term follow-up unavailable

This is a single anonymised case report. Formal IRB approval was not required by the institutional review board of Prince Hamzah Hospital, Amman, Jordan, as the study involved no experimental intervention, no prospectively collected research data, and full anonymisation of all patient information. Verbal consent for treatment and open access publication was obtained from the patient, and the requirement for written consent was waived by the institution. No patient identifying information appears in the text or images.

## Discussion

This case demonstrates that airway risk in deep neck infection must be assessed dynamically and comprehensively rather than determined solely by oxygen saturation, dyspnoea, or the presence of stridor. The patient maintained spontaneous ventilation on room air and had no stridor, yet she demonstrated multiple features that collectively elevate anaesthetic airway risk: muffled voice, documented difficulty speaking, odynophagia, dysphagia, pain-limited oral examination, mild supine discomfort, progressive multi-compartment neck infection, CT-documented SCM involvement, and confirmed tracheal deviation. The recognition of these features - not the baseline SpO₂ - appropriately drove the level of preparation in this case.

The first key lesson is that a clinically stable airway can deteriorate precipitously after induction of anaesthesia. A deep neck infection progressively distorts upper-airway anatomy through oedema, tongue elevation, and supraglottic involvement, without necessarily producing overt stridor until obstruction is near-complete [5]. Muffled voice quality reflects oropharyngeal or hypopharyngeal involvement and has been associated with increased airway difficulty [5]. Supine discomfort and dysphagia indicate pharyngeal and potentially supraglottic compromise that may worsen after induction. In Ludwig's angina specifically, progressive tongue displacement can impair mask ventilation and laryngeal visualisation even in patients who appear to manage their airway adequately while upright [1,7].

The second lesson concerns the choice between awake and asleep airway management. Awake tracheal intubation, typically performed by flexible bronchoscopy under topical anaesthesia, is regarded as the most reliable technique when difficult intubation, difficult mask ventilation, aspiration risk, poor apnoea tolerance, or difficult rescue airway access is anticipated [1,2]. The Difficult Airway Society guidelines specifically highlight that awake tracheal intubation is underused in anticipated difficult-airway scenarios, emphasising its high success rate and favourable safety profile [2]. In the present case, awake tracheal intubation was not performed; the team elected to proceed with C-MAC video-laryngoscopic assessment in the operating room, with the otolaryngology surgeon immediately available and tracheostomy equipment prepared. This approach was successful, with a grade 1 view and first-attempt intubation achieved. However, this result represents a successful individualised decision made under emergency conditions and should not be generalised as a recommendation that asleep video-laryngoscopic management is equivalent or superior to awake techniques in similar infections [1,2,5].

The third lesson concerns surgical airway readiness. Even when intubation proves uncomplicated, the presence of an otolaryngology surgeon and prepared tracheostomy equipment at induction provides an irreplaceable safety margin. Emergency front-of-neck access in Ludwig's angina may itself be technically hazardous owing to tissue oedema, infection, landmark distortion, and a contaminated surgical field. Pre-induction multidisciplinary planning and physical readiness for a surgical airway are therefore non-negotiable elements of safe management, as reflected in current difficult-airway guidelines [1].

The fourth lesson is that extubation after deep neck infection drainage is itself a high-risk airway event requiring structured planning. Residual oedema, ongoing infection, and surgical manipulation may make immediate extubation unsafe after initial drainage. In this case, the patient was maintained intubated in the ICU, and extubation was performed approximately 24 hours after surgery as a planned procedure, incorporating cuff-leak testing, corticosteroid pre-treatment, multidisciplinary team presence, and a prepared reintubation plan. This approach directly reflects the Difficult Airway Society extubation guideline, which classifies such patients as at-risk and requires a structured extubation strategy and post-extubation monitoring plan [6].

The fifth lesson is that the requirement for a second anaesthetic in this admission - and its successful conduct with a grade 1 direct laryngoscopy view and immediate extubation - illustrates two principles: first, that residual or recurrent collections after initial deep neck infection drainage are a recognised clinical phenomenon requiring surgical re-exploration [8,9]; and second, that each return to the operating room requires independent airway reassessment, not reliance on previous intubation findings.

Finally, the microbiological finding of S. agalactiae as the isolate from this odontogenic deep neck abscess is clinically interesting. Group B Streptococcus is most commonly associated with neonatal sepsis and adult invasive infections in immunocompromised or elderly patients; its recovery in an immunocompetent adult from an odontogenic deep neck infection, while unusual, has been reported [9]. The polymicrobial nature of odontogenic infections means that a single aerobic culture does not fully characterise the microbial flora [5,7-9]. The susceptibility profile - resistant to clindamycin and erythromycin, susceptible to penicillin, amoxicillin/clavulanate, levofloxacin, and vancomycin - was consistent with the antimicrobial agents already prescribed, supporting the decision not to alter therapy after culture results.

## Conclusions

Odontogenic deep neck space infection managed clinically as Ludwig's angina presents a complex and dynamic airway challenge that demands structured perioperative planning at every stage. Absence of stridor or hypoxaemia does not exclude significant anaesthetic airway risk. In this case, muffled voice, documented difficulty speaking, pain-limited oral examination, mild supine discomfort, multi-compartment neck collections, sternocleidomastoid involvement, and confirmed tracheal deviation together justified a high level of preparation. C-MAC video-laryngoscopic assessment in the operating room with otolaryngology on standby and prepared tracheostomy equipment was the strategy selected in this emergency setting. Planned extubation with cuff-leak testing, corticosteroid pre-treatment, and multidisciplinary team presence was performed in the ICU approximately 24 hours postoperatively. A second anaesthetic was required five days later for repeat drainage, again achieving uncomplicated intubation and immediate extubation. The total duration of the antimicrobial course was 21 days. The case reinforces that airway risk in deep neck infection requires repeated, multi-factorial reassessment throughout the clinical course and that each anaesthetic encounter - including extubation - should be treated as a planned, potentially difficult-airway procedure.

## References

[1] Apfelbaum JL, Hagberg CA, Connis RT (2022). 2022 American Society of Anesthesiologists practice guidelines for management of the difficult airway. Anesthesiology.

[2] Ahmad I, El-Boghdadly K, Bhagrath R (2020). Difficult Airway Society guidelines for awake tracheal intubation (ATI) in adults. Anaesthesia.

[3] Riley DS, Barber MS, Kienle GS (2017). CARE guidelines for case reports: explanation and elaboration document. J Clin Epidemiol.

[4] Hendrix JM, Garmon EH (2025). American Society of Anesthesiologists classification. StatPearls [Internet].

[5] Dowdy RA, Emam HA, Cornelius BW (2019). Ludwig's angina: anesthetic management. Anesth Prog.

[6] Popat M, Mitchell V, Dravid R, Patel A, Swampillai C, Higgs A (2012). Difficult Airway Society Guidelines for the management of tracheal extubation. Anaesthesia.

[7] AL Ghabra Y, Brizuela M, Winters R, Singhal M (2025). Ludwig angina. StatPearls [Internet].

[8] Almuqamam M, Gonzalez FJ, Kondamudi NP (2024). Deep neck infections. StatPearls [Internet].

[9] Bridwell R, Gottlieb M, Koyfman A, Long B (2021). Diagnosis and management of Ludwig's angina: an evidence-based review. Am J Emerg Med.

